# Serum Perfluorooctanoate (PFOA) and Perfluorooctane Sulfonate (PFOS) Concentrations and Liver Function Biomarkers in a Population with Elevated PFOA Exposure

**DOI:** 10.1289/ehp.1104436

**Published:** 2012-01-30

**Authors:** Valentina Gallo, Giovanni Leonardi, Bernd Genser, Maria-Jose Lopez-Espinosa, Stephanie J. Frisbee, Lee Karlsson, Alan M. Ducatman, Tony Fletcher

**Affiliations:** 1 Social and Environmental Health Research, London School of Hygiene and Tropical Medicine, London, United Kingdom; 2 School of Public Health, Imperial College London, London, United Kingdom; 3 Mannheim Institute of Public Health, Social and Preventive Medicine, University of Heidelberg, Heidelberg, Germany; 4 Instituto de Saúde Coletiva, Federal University of Bahia, Salvador, Brazil; 5 Department of Community Medicine and Center for Cardiovascular and Respiratory Sciences, and; 6 Department of Community Medicine, West Virginia University School of Medicine, Morgantown, West Virginia, USA

**Keywords:** C8, cross-sectional study, liver function biomarkers, PFOA, PFOS, population-based survey

## Abstract

Background: Perfluorooctanoate (PFOA) and perfluorooctane sulfonate (PFOS) persist in the environment and are found in relatively high concentrations in animal livers. Studies in humans have reported inconsistent associations between PFOA and liver enzymes.

Objectives: We examined the cross-sectional association between serum PFOA and PFOS concentrations with markers of liver function in adults.

Methods: The C8 Health Project collected data on 69,030 persons; of these, a total of 47,092 adults were included in the present analysis. Linear regression models were fitted for natural log (ln)-transformed values of alanine transaminase (ALT), γ-glutamyltransferase (GGT), and direct bilirubin on PFOA, PFOS, and potential confounders. Logistic regression models were fitted comparing deciles of PFOA or PFOS in relation to high biomarker levels. A multilevel analysis comparing the evidence for association of PFOA with liver function at the individual level within water districts to that at the population level between water districts was also performed.

Results: ln-PFOA and ln-PFOS were associated with ln-ALT in linear regression models [PFOA: coefficient, 0.022; 95% confidence interval (CI): 0.018, 0.025; PFOS: coefficient, 0.020; 95% CI: 0.014, 0.026] and with raised ALT in logistic regression models [with a steady increase in the odds ratio (OR) estimates across deciles of PFOA and PFOS; PFOA: OR = 1.10; 95% CI: 1.07, 1.13; PFOS: OR = 1.13; 95% CI: 1.07, 1.18]. There was less consistent evidence of an association of PFOA and GGT or bilirubin. The relationship with bilirubin appears to rise at low levels of PFOA and to fall again at higher levels.

Conclusions: These results show a positive association between PFOA and PFOS concentrations and serum ALT level, a marker of hepatocellular damage.

Perfluorooctanoate (PFOA) and perfluoro-octane sulfonate (PFOS) are two members of the perfluoroalkyl acid (PFAA) class of chemicals, man-made compounds used in the manufacture of fluoro-polymers, including those used for non-stick cookware and breathable, waterproof fabrics. PFOA and PFOS can also result from the metabolism of fluori-nated telomers, compounds used for food package coatings, carpet treatments, and stain-resistant fabric treatment. PFOA and PFOS persist in the environment (Kato et al. 2011; Tao et al. 2006); potential sources of exposure to PFOA and PFOS in humans include drinking water, dust, breast milk, food packaging, ambient air, and occupation (D’Hollander et al. 2010; Goosey and Harrad 2011; Kim et al. 2011; Lau et al. 2007).

In rodents and non-human primates, both PFOA and PFOS have been found in relatively high concentrations in the liver and have been associated with liver enlargement. In rats, these compounds have been also associated with hepatocellular adenomas (Lau et al. 2007). In mice, one of the biological effects of PFAAs is the activation of the peroxisome proliferator-activated receptor-α (PPAR-α), a ligand--activated transcription factor that regulates gene expression, lipid modulation, glucose homeostasis, cell proliferation, and inflammation (Pyper et al. 2010). Although some effects in experimental studies are mediated by PPAR-α binding, some other effects occur independently of this receptor (Bjork et al. 2011; Ren et al. 2009). PPAR-α is also induced by PFOA and PFOS in transiently transfected human fibroblast-like cell line COS-1, in a concentration-dependent and in a roughly chain-length–dependent fashion (Wolf et al. 2008).

Studies in humans have reported inconsistent associations between PFOA or PFOS and liver enzymes. Transaminase levels have been positively associated with PFOA concentrations in some occupational studies (Costa et al. 2009; Olsen and Zobel 2007; Sakr et al. 2007b) but not in others (Costa et al. 2009; Sakr et al. 2007b). Similarly, γ-glutamyltransferase (GGT) levels have been inconsistently associated with PFOA concentrations in occupational studies (Costa et al. 2009; Olsen and Zobel 2007; Sakr et al. 2007a, 2007b). In a large population-based survey, PFOA but not PFOS was associated with both transaminase and GGT levels (Lin et al. 2010), although those findings differed from those of an earlier, much smaller population-based study of subjects heavily exposed to PFOA (Emmett et al. 2006). Direct bilirubin has been found to be negatively associated with PFOA concentrations in a few occupational studies (Costa et al. 2009; Olsen and Zobel 2007; Sakr et al. 2007b) but not in others (Costa et al. 2009; Sakr et al. 2007a). No association between direct bilirubin and PFOA or PFOS was observed in population-based studies (Emmett et al. 2006; Lin et al. 2010).

From 1950 through 2005, a chemical plant in the Mid-Ohio Valley, West Virginia (USA), was responsible for emitting PFOA into the surrounding environment. In 2001, a group of residents filed a class action lawsuit alleging health damage from the drinking water supplies drawing on PFOA-contaminated groundwater (Frisbee et al. 2009). Part of the pre-trial settlement of the class action lawsuit included a baseline survey, the C8 Health Project, conducted in 2005–2006, that gathered data from > 69,000 persons from six contaminated water districts surrounding the plant (Frisbee et al. 2009). In this population, overall PFOA levels were much higher [mean, 83.0 ng/mL; interquartile range (IQR), 13.4–70.6 ng/mL] than in corresponding U.S. population surveys (National Health and Nutrition Examination Survey in the same year: mean, 3.9 ng/mL; IQR, 2.7–5.8 ng/mL) (Frisbee et al. 2009; Kato et al. 2011). However, the mean PFOS (23.3 ng/mL; IQR, 13.8–29.0 ng/mL) closely resembled the U.S. population mean (20.7 ng/mL; IQR, 14.6–29.9 ng/mL) (Kato et al. 2011). The present study used these data to examine the cross-sectional association between serum PFOA or PFOS concentrations and markers of liver function in adults.

## Methods

*The study population.* This study was approved by the London School of Hygiene and Tropical Medicine Ethics Committee and is one of the C8 Science Panel studies and used information from questionnaires and blood tests collected in the C8 Health Project, supplemented by further information on classification by water district developed in a companion C8 Science Panel study (Shin et al. 2011).

The C8 Health Project enrolled eligible subjects between August 2005 and August 2006. Individuals were eligible to partici-pate if they had consumed water for at least 1 year between 1950 and 2004 while living, working, or going to school in one of the six water districts, or private water sources, or areas of documented PFOA contamina-tion. The between- and within-group regression analysis was restricted to subjects living in one of the six contaminated water districts at the time of survey [for additional details on water districts, see Supplemental Material (http://dx.doi.org/10.1289/ehp.1104436)]. Details of the study enrollment process, including consenting procedures, have been described elsewhere (Frisbee et al. 2009).

The C8 Health Project collected data on 69,030 persons. Its participation rate, based on U.S. census numbers, has been estimated at around 80% (Frisbee et al. 2009). In this population, the strongest predictor of PFOA serum concentration was residence in one of the contaminated water districts (Steenland et al. 2009), whereas serum levels of other PFAAs did not show such geographic variation. Of the population, 56,554 adults (≥ 18 years of age) were considered for this analysis, and a total of 46,452 of those adults (82.1%) were included in the final analysis after exclusion of subjects with missing data on socioeconomic status, alcohol consumption, or cigarette smoking or other potential confounding variables or without PFAAs or liver enzymes measurements.

*Choice of parameters and laboratory analyses.* Blood samples were obtained and processed at individual data collection sites. Samples were drawn into four tubes per participant, with a maximum of 35 mL blood collected. Samples were centrifuged, aliquoted, and refrigerated until shipping. Processed samples were shipped on dry ice daily from each data collection site to the laboratory (Frisbee et al. 2009). Participants were not asked to fast before blood sample withdrawal, but fasting status was recorded.

Laboratory analyses of PFAAs were conducted by the Exygen Research Inc. (State College, PA, USA). using an automated solid-phase extraction combined with reverse-phase high-performance liquid chromatography/mass spectrometry (Kuklenyik et al. 2004). An intralaboratory quality assurance program was carried out by analysis of duplicate samples at AXYS Analytical Service Ltd. (Sidney, BC, Canada) (Frisbee et al. 2009). The intralaboratory coefficient of variation for both PFOA and PFOS measurements was 0.1; the interlaboratory comparison coefficient of variation was 0.2 for PFOA and 0.1 for PFOS (Frisbee et al. 2009). The detection limit was 0.5 ng/mL for both PFOA and PFOS, and observations below this limit were assigned a value of 0.25 ng/mL (for this study population, *n* = 32 for PFOA, *n* = 230 for PFOS). Both PFOA and PFOS concentration distributions were skewed to the right.

The liver parameters we measured were alanine aminostransferase (ALT) and aspartate aminostransferase (AST), GGT, alkaline phosphatase (ALP), and direct bilirubin (also known as “conjugated bilirubin”). Both transaminases (AST and ALT) are enzymes released after liver parenchymal cell injury and are elevated in serum during acute liver damage; the correlation between ALT and AST in the present population is *r* = 0.79. To limit multiple comparisons and to be consistent with the most recent published literature on the same topic (Lin et al. 2010), we restricted our analysis to ALT, GGT, and direct bilirubin as markers of liver function. Elevated ALT has been used as a proxy for hepatocellular injury in previous studies (Clark et al. 2003; Ioannou et al. 2005; Lin et al. 2010; Ruhl and Everhart 2003) because it is more specific for hepatic damage than is AST. Elevation of GGT occurs at an early stage and is more persistent than that of ALP in cholestatic disorders (Whitfield 2001); the correlation between GGT and ALP in the present population was *r* = 0.29. Bilirubin is mostly derived from the metabolism of hemoglobin; the increase in serum of the direct bilirubin component is highly specific for liver or bile duct disease (Sedlak and Snyder 2004).

ALT, GGT, and direct bilirubin were measured using a Roche/Hitachi MODULAR automated analyzer (Roche Diagnostics, Indianapolis, IN, USA), and the analyses were performed at a large, independent, accredited clinical diagnostic laboratory (LabCorp, Inc., Burlington, NC, USA) (Frisbee et al. 2009). The homeostasis model assessment of insulin resistance (HOMA-IR) index was calculated as the product of basal glucose and insulin levels divided by 2.25; it is used as a surrogate measure for insulin resistance (Matthews et al. 1985).

Methods and results are described according to the STrengthening the Reporting of OBservational studies in Epidemiology—Molecular Epidemiology (STROBE-ME) guidelines (Gallo et al. 2011).

*Statistical analysis.* Distributions of continuous variables were inspected, and all three outcome markers were natural log (ln) transformed for linear regression models. We investigated univariate associations between exposure (serum concentration of PFOA and PFOS), liver function biomarkers (ALT, GGT, direct bilirubin), and potential confounders: age, physical activity, body mass index (BMI; classified as underweight/normal weight/overweight, obese class I/II/III), average household income (≤ $10,000, $10,001–20,000, $20,001–30,000, $30,001–40,000, $40,001–50,000, $50,001–60,000, $60,001–70,000, > $70,000), educational level [high school diploma or general educational development (GED), some college, bachelor degree or higher], race (white, black, other), alcohol consumption (none, < 1 drink/month, < 1 drink/week, few drinks/week, 1–3 drinks/day, > 3 drinks/day) and cigarette smoking (never smoker, former, current < 10 cigarettes/day, current 10–19 cigarettes/day, current ≥ 20 cigarettes/day).

The association between ALT, GGT, or direct bilirubin and PFOA or PFOS was assessed using linear regression models. First, we fitted a simple model including only age and sex (model 1), followed by a model additionally including alcohol consumption, socioeconomic status, fasting status, race, and month of blood sample collection (model 2), and then a model additionally including smoking status, BMI, physical activity, and insulin resistance (i.e., HOMA-IR) in deciles of distribution (model 3).

Also, specific cutoff values were used to fit logistic regression models to estimate the impact of PFOA and PFOS on being above these values. Cutoff values used were 45 IU/L in men and 34 IU/L in women for ALT (Schumann et al. 2002a), giving a total of 5,194 persons (11.2% of the 47,092 eligible participants) with above-normal values; 55 IU/L in men and 38 IU/L in women for GGT (Schumann et al. 2002b), giving 5,990 persons (12.9%) with above-normal values; and 0.3 mg/dL in men and women for direct bilirubin (Kratz et al. 2004), giving 506 persons (1.1%) with above-normal values.

Prior research showed that individual serum levels of PFOA were strongly associated with the water district of residence (Steenland et al. 2009). This geographic clustering implies a potential for an ecological confounding by other uncontrolled and/or unobserved water district–specific factors. On the other hand, there is also potential for confounding at the individual level. We expect *a priori* some heterogeneity in between- and within-group relationships; therefore, we applied an analytic approach in the multilevel framework [between- and within-group regression (Davis et al. 1961)] to disentangle between- and within-district effects of PFOA. In statistical modeling, this is realized by simultaneously incorporating both individual PFOA serum concentrations and the means of PFOA within water districts. Details of this analysis are reported in the Supplemental Material (http://dx.doi.org/10.1289/ehp.1104436).

## Results

General characteristics of the population are summarized in Table 1. Women had significantly lower values of liver function biomarkers and of PFOA and PFOS. PFOA and PFOS concentrations were associated with all potential confounders considered.

**Table 1:** Participant characteristics, Mid-Ohio Valley, 2005–2006.

Characteristic	Women (n = 24,171)	Men (n = 22,281)	Total (n = 46,452)	p-Valuea
Age (median/mean ± SD)		44.3/45.1 ± 15.9		45.5/45.8 ± 15.8		44.7/45.5 ± 15.9		< 0.001
Regular exercise [n (%)]		7,821 (32.4)		6,741 (30.3)		14,562 (31.4)		< 0.001
BMI [n (%)]								
Underweight		471 (2.0)		153 (0.7)		624 (1.3)		< 0.001
Normal weight		8,236 (34.1)		5,156 (23.1)		13,392 (28.8)		
Overweight		6,848 (28.3)		9,339 (41.9)		16,187 (34.9)		
Obese, class I		4,545 (18.8)		5,066 (22.7)		9,611 (20.7)		
Obese, class II		2,257 (9.3)		1,753 (7.9)		4,010 (8.6)		
Obese, class III		1,814 (7.5)		814 (3.7)		2,628 (5.7)		
Household income, US$/year [n (%)]								
≤ 10,000		2,852 (11.8)		2,081 (9.3)		4,933 (10.6)		< 0.001
10,001–20,000		4,127 (17.1)		3,018 (13.6)		7,145 (15.4)		
20,001–30,000		3,899 (16.1)		3,636 (16.3)		7,535 (16.2)		
30,001–40,000		3,340 (13.8)		3,265 (14.7)		6,605 (14.2)		
40,001–50,000		2,690 (11.1)		2,664 (12.0)		5,354 (11.5)		
50,001–60,000		2,207 (9.1)		2,277 (10.2)		4,484 (9.7)		
60,001–70,000		1,736 (7.2)		1,817 (8.2)		3,553 (7.7)		
> 70,000		3,320 (13.7)		3,523 (15.8)		6,843 (14.7)		
Education [n (%)]								
< 12 years		2,630 (10.9)		2,623 (11.8)		5,253 (11.3)		< 0.001
High school diploma or GED		9,453 (39.1)		9,935 (44.6)		19,388 (41.7)		
Some college		8,760 (36.2)		6,723 (30.2)		15,483 (33.3)		
≥ Bachelor degree		3,328 (13.8)		3,000 (13.5)		6,328 (13.6)		
Race [n (%)]								
White		23,531 (97.4)		21,685 (97.3)		45,216 (97.3)		0.009
Black		209 (0.9)		245 (1.1)		454 (1.0)		
Other		431 (1.8)		351 (1.6)		782 (1.7)		
Alcohol consumption [n (%)]								
None		13,487 (55.8)		9,968 (44.7)		23,455 (50.5)		< 0.001
< 1 drink/month		5,251 (21.7)		3,128 (14.0)		8,379 (18.0)		
< 1 drink/week		2,797 (11.6)		2,717 (12.2)		5,514 (11.9)		
Few drinks/week		2,209 (9.1)		4,630 (20.8)		6,839 (14.7)		
1–3 drinks/day		336 (1.4)		1,225 (5.5)		1,561 (3.4)		
> 3 drinks/day		91 (0.4)		613 (2.8)		704 (1.5)		
Smoking status [n (%)]								
Never		12,775 (52.9)		9,489 (42.6)		22,264 (47.9)		< 0.001
Former		5,178 (21.4)		6,998 (31.4)		12,176 (26.2)		
Current < 10 cigarettes/day		2,836 (11.7)		1,900 (8.5)		4,736 (10.2)		
Current 10–19 cigarettes/day		2,285 (9.5)		2,124 (9.5)		4,409 (9.5)		
Current ≥ 20 cigarettes/day		1,097 (4.5)		1,770 (7.9)		2,867 (6.2)		
ALT (IU/L)								
Median/mean ± SD		17.0/20.8 ± 16.0		26/31.0 ± 22.5		21.0/25.7 ± 20.1		< 0.001
≤ 45 (m), ≤ 34 (w) [n (%)]		22,088 (91.4)		19,170 (86.0)		41,258 (88.8)		< 0.001
> 45 (m), > 34 (w) [n (%)]		2,083 (8.6)		3,111 (14.0)		5,194 (11.2)		
GGT (IU/L)								
Median/mean ± SD		17.0/24.3 ± 31.0		27.0/37.5 ± 53.5		21.0/30.7 ± 43.7		< 0.001
≤ 55 (m), ≤ 38 (w) [n (%)]		21,243 (87.9)		19,219 (86.3)		40,462 (87.1)		< 0.001
> 55 (m), > 38 (w) [n (%)]		2,928 (12.1)		3,062 (13.7)		5,990 (12.9)		
Direct bilirubin (mg/dL)								
Median/mean ± SD		0.1/0.1 ± 0.4		0.1/0.1 ± 0.6		0.1/0.1 ± 0.6		< 0.001
≤ 0.3 [n (%)]		24,045 (99.5)		21,901 (98.3)		45,946 (98.9)		< 0.001
> 0.3 [n (%)]		126 (0.5)		380 (1.7)		506 (1.1)		
PFOA, ng/mL [median (IQR)]		23.1 (11.3–58.2)		34.3 (16.6–85.1)		28.0 (13.5–70.8)		< 0.001
PFOS, ng/mL [median (IQR)]		17.4 (11.6–25.5)		23.5 (16.8–32.6)		20.3 (13.7–29.4)		< 0.001
Abbreviations: m, men; w, women. ap-Value for chi-square test for categorical variables, and t-test for continuous variables–based testing the difference between sexes.								

ln-Transformed values of ALT were significantly associated with ln-PFOA and ln-PFOS in linear regression models [fully adjusted (model 3) coefficient: PFOA, 0.022; 95% confidence interval (CI): 0.018, 0.025; PFOS, 0.020; 95% CI: 0.014, 0.026], with a partial *R*^2^ greater for the association with PFOA (partial *R*^2^ = 0.002) than for that with PFOS (partial *R*^2^ < 0.001; Table 2). In Figure 1, mean ALT levels are plotted against deciles of PFOA and PFOS concentrations, adjusting for covariates by setting these to their means. A steady increase in fitted levels of ALT per decile of PFOA is shown, with a possible leveling off effect after approximately 30 ng/mL. A comparable distribution is observable for PFOS, although in a narrower range of exposure (Figure 1A,B). This positive association was also observed in logistic regression models with a steady increase in the odds ratio (OR) estimates across deciles of both PFOA and PFOS (*p*-value for trends across deciles in both models < 0.001), and a significant OR for both ln-unit of PFOA (OR = 1.10; 95% CI: 1.07, 1.13) and ln-unit of PFOS (OR = 1.13; 95% CI: 1.07, 1.18; Table 3). Subjects with abnormally high ALT values were evenly distributed across water districts (chi-square test, *p* = 0.172) and had significantly higher PFOA and PFOS serum concentrations compared with those with normal ALT values (PFOA: 106.8 vs. 84.6 IU/L, *p* < 0.001; PFOS: 24.3 vs. 23.3 IU/L, *p* < 0.001). No interaction between PFOA/PFOS deciles with fasting status was observed (*p*-values 0.816 and 0.387, -respectively).

**Table 2:** Linear regression coefficients of ln-transformed blood analytes with a one-unit increase in ln-PFOA and ln-PFOS concentrations.

ln-PFOA			ln-PFOS		
Liver function biomarker	Coefficient (95% CI)	R2 (partial R2)a	Coefficient (95% CI)	R2 (partial R2)a
ln-ALT								
Model 1b		0.018 (0.014, 0.021)**		0.170 (0.002)		0.029 (0.023, 0.036)**		0.170 (0.002)
Model 2c		0.014 (0.010, 0.018)**		0.174 (0.001)		0.026 (0.020, 0.033)**		0.175 (0.002)
Model 3d		0.022 (0.018, 0.025)**		0.265 (0.002)		0.020 (0.014, 0.026)**		0.263 (< 0.001)
ln-GGT								
Model 1b		0.005 (–0.0001, 0.009)		0.145 (< 0.001)		–0.008 (–0.016, –0.001)		0.145 (< 0.001)
Model 2c		0.004 (–0.001, 0.009)		0.166 (< 0.001)		0.006 (–0.002, 0.014)		0.166 (< 0.001)
Model 3d		0.015 (0.010, 0.019)**		0.249 (0.001)		0.008 (–0.0002, 0.016)		0.248 (< 0.001)
ln-Direct bilirubin								
Model 1b		0.004 (0.001, 0.007)*		0.094 (< 0.001)		0.033 (0.028, 0.039)**		0.097 (0.003)
Model 2c		0.003 (0.0004, 0.006)*		0.121 (< 0.001)		0.034 (0.029, 0.040)**		0.124 (0.003)
Model 3d		0.001 (–0.002, 0.004)		0.163 (< 0.001)		0.029 (0.024, 0.034)**		0.166 (0.003)
aPartial R2, difference between R2 including and excluding PFOA or PFOS in the model. bAdjusted for age and sex. cAdjusted for alcohol consumption, socioeconomic status, fasting status, race, and month of blood sample collection in addition to adjustment in model 1. dAdjusted for smoking status, BMI, physical activity, and insulin resistance in addition to adjustment in model 2. *p < 0.05. **p < 0.001.								

**Figure 1:**
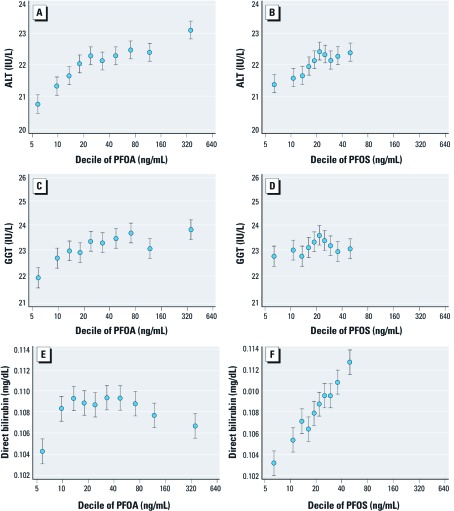
Fitted values of ALT (*A*,*B*), GGT (*C*,*D*), and direct bilirubin (*E*,*F*) levels (mean and 95% CI, from fully adjusted regression model) by deciles of PFOA (*A*,*C*,*E*) and PFOS (*B*,*D*,*F*) concentrations, given the mean values of the other covariates. Graph pairs are on the same scale.

**Table 3:** Logistic regression ORa (95% CI) of having abnormal high values of ALT, GGT, or direct bilirubin across deciles of PFOA and PFOS.

Decile	ALT	GGT	Direct bilirubin	Decile	ALT	GGT	Direct bilirubin
PFOA								PFOS						
Decile 1		Reference		Reference		Reference		Decile 1		Reference		Reference		Reference
Decile 2		1.09 (0.94, 1.26)		1.06 (0.93, 1.21)		1.01 (0.66, 1.54)		Decile 2		1.01 (0.87, 1.16)		1.06 (0.94, 1.20)		0.75 (0.47, 1.20)
Decile 3		1.19 (1.03, 1.37)		1.07 (0.94, 1.21)		1.04 (0.69, 1.56)		Decile 3		1.06 (0.91, 1.22)		0.95 (0.83, 1.07)		0.62 (0.39, 1.01)
Decile 4		1.26 (1.09, 1.45)		1.05 (0.93, 1.20)		0.95 (0.62, 1.44)		Decile 4		1.11 (0.96, 1.28)		0.93 (0.82, 1.06)		0.72 (0.46, 1.13)
Decile 5		1.40 (1.22, 1.62)		1.11 (0.97, 1.26)		0.89 (0.58, 1.37)		Decile 5		1.19 (1.04, 1.37)		0.96 (0.84, 1.09)		0.85 (0.55, 1.31)
Decile 6		1.39 (1.21, 1.60)		1.10 (0.96, 1.25)		1.03 (0.68, 1.56)		Decile 6		1.19 (1.04, 1.37)		1.03 (0.91, 1.17)		0.95 (0.63, 1.44)
Decile 7		1.31 (1.14, 1.52)		1.20 (1.06, 1.37)		0.86 (0.56, 1.32)		Decile 7		1.20 (1.04, 1.38)		0.97 (0.85, 1.10)		1.01 (0.67, 1.52)
Decile 8		1.42 (1.23, 1.64)		1.13 (0.99, 1.29)		0.95 (0.62, 1.45)		Decile 8		1.24 (1.08, 1.43)		0.91 (0.80, 1.04)		0.93 (0.61, 1.41)
Decile 9		1.40 (1.21, 1.62)		1.06 (0.93, 1.22)		0.80 (0.52, 1.25)		Decile 9		1.18 (1.02, 1.36)		0.89 (0.78, 1.01)		0.91 (0.60, 1.39)
Decile 10		1.54 (1.33, 1.78)		1.06 (0.92, 1.20)		1.01 (0.66, 1.53)		Decile 10		1.25 (1.08, 1.44)		0.94 (0.83, 1.07)		1.23 (0.82, 1.83)
Trendb		< 0.001		0.213		0.496		Trendb		< 0.001		0.047		0.015
ln-Unitc		1.10 (1.07, 1.13)		1.01 (0.99, 1.04)		0.97 (0.90, 1.05)		ln-unitc		1.13 (1.07, 1.18)		0.98 (0.94, 1.02)		1.11 (0.96, 1.28)
p-Valuec		< 0.001		0.298		0.512		p-Valuec		< 0.001		0.310		0.168
aAdjusted by age, sex, alcohol consumption, socioeconomic status, fasting status, month of blood sample collection, smoking, BMI, physical activity, increased serum iron, and insulin resistance. bp-Value for trend across deciles. cOR and relative 95% CI and p-value for ln-PFOA/PFOS.														

The association between ln-GGT and ln-PFOA reached a significant level in the fully adjusted model (model 3; coefficient, 0.015; 95% CI: 0.010, 0.019) mainly due to the contribution of insulin resistance and BMI, which appeared to be highly associated with ln-GGT (Table 2); fitted values of GGT by deciles of PFOA showed an apparent positive association (Figure 1C), although it was less clear than that shown for ALT. The suggested association with PFOA, however, was not confirmed in the logistic regression model, in which no trend across deciles was observed (*p* = 0.213), or for the linear ln-units of PFOA values (OR = 1.01; 95% CI: 0.99, 1.04; Table 3). For PFOS, there was some evidence of a slight inverse association with GGT in the minimally adjusted linear regression model (model 1), which was lost after adjusting for additional confounders (model 3; Table 2). The logistic regression by deciles of PFOS suggested a weak negative trend across deciles, although all ORs were close to 1, and no overall association with ln-PFOS was observed (Table 3). Overall, fitted values of GGT were unchanged across deciles of PFOS (Figure 1D). Subjects with abnormally high GGT values were more frequent in one district (chi-square test, *p* = 0.062) and had significantly higher PFOA serum concentrations (93.7 vs. 86.1 IU/L, *p* = 0.004) but not PFOS serum concentrations (23.5 vs. 23.4 IU/L, *p* = 0.499).

For direct bilirubin, there was a suggestion of an inverse U-shaped relationship with PFOA, with increasing levels of bilirubin per increasing levels of PFOA at low PFOA levels, and decreasing bilirubin levels for concentrations of PFOA above about 40 ng/mL (Figure 1E). Overall, the linear regression relationship failed to show any association in the adjusted model (Table 2), and the likelihood ratio test after introducing the quadratic term was statistically significant (*p* < 0.001). In accordance, no pattern was evident in the logistic regression models of high bilirubin (Table 3). By contrast, for PFOS, a clear positive association was shown in linear regression models for direct bilirubin (coefficient, 0.029; 95% CI: 0.024, 0.034; Table 2, Figure 1F), although this was not evident in logistic regression models (Table 3). However, logistic regression results for direct bilirubin should be interpreted cautiously because only 1.1% of the whole sample had high levels and CIs were wide.

**Figure 2:**
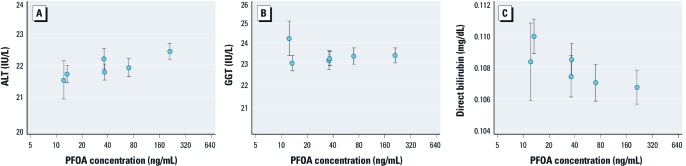
Fitted values of ALT (*A*), GGT (*B*), and direct bilirubin (*C*) levels (mean and 95% CI; from fully adjusted regression model) by mean PFOA concentration in the water district, given the mean values of the other covariates.

Multilevel analysis was restricted to subjects living in water districts supplied by contaminated water (*n* = 26,777) and excluding those with private wells. The fitted values for each of the outcomes of ALT, GGT, and direct bilirubin versus mean PFOA serum level for the six water districts are graphed in Figure 2. In Table 4, the regression coefficients per ln-unit of PFOA for a model between water districts comparing district averages of ln-PFOA (*B*) and for a model within water districts comparing differences from the average (*W*) are presented. There was a significant difference between the between- and within-district components (*p*-value *B*/*W*Δ) for ALT and direct bilirubin; however, each outcome showed different patterns. The between--water-district regression coefficient from linear regression of ln-PFOA and ALT (0.010; 95% CI: –0.001, 0.020) was lower than the within-water-district coefficient (0.027; 95% CI: 0.022, 0.031). However, both coefficients were significant or borderline significant, in the same direction, and consistent with a positive association between ALT and PFOA levels. Although the regression coefficient for the association between GGT and ln-PFOA within water district was positive and significant (0.016; 95% CI: 0.010, 0.023), this association was not significant in the between-water-district analysis (0.005; 95% CI: –0.009, 0.018); the *p*-value for interaction between/within water district was not significant (*p* = 0.108). Conversely, there was a significant inverse relationship between ln-PFOA and direct bilirubin between water districts (–0.013; 95% CI: –0.022, –0.005) but not within water districts (0.0001; 95% CI: –0.004, 0.004).

**Table 4:** Multilevel analysis of PFOA between and within district effects.

Liver function biomarker	Coefficient between districts (95% CI)	Coefficient within districts (95% CI)	p-Valuea
ln-PFOA						
ALT		0.010 (–0.001, 0.020)#		0.027 (0.022, 0.031)**		0.003
GGT		0.005 (–0.009, 0.018)		0.016 (0.010, 0.023)**		0.108
Direct bilirubin		–0.013 (–0.022, –005)*		0.0001 (–0.004, 0.004)		0.004
ap-Value testing the null hypothesis of no difference between and within water district. *p < 0.05. **p < 0.001. #p = 0.062.						

## Discussion

These results show a positive association between PFOA and PFOS concentrations and ALT serum levels, a marker of hepatocellular damage. The linear association, consistently replicated in all analyses, showed a monotonic increase in logistic regression. Furthermore, the presence of a consistent relationship in regression analysis both between water districts and among individuals within districts increases strength of evidence for causal association. The proportion of laboratory abnormal values rises in relation to PFOA, but the small amount of variation in outcomes explained by PFOA (partial *R*^2^ ≤ 0.1%) suggests caution.

The observed associations between PFOA or PFOS and ALT are generally consistent in terms of direction and magnitude with previous findings of occupational studies. A cross-sectional study of 1,025 workers at the same plant leading to the C8 Health Project reported an association between PFOA and ALT levels with a similar coefficient (mean ± SE coefficient of log-transformed values, 0.023 ± 0.015; *p* = 0.124). A significant linear regression coefficient between log-transformed–PFOA and log-ALT (coefficient, 0.025 ± 0.013; *p* = 0.006) was described in a cross-sectional occupational surveillance conducted in three 3M plants in the United States and Belgium (Olsen and Zobel 2007). A small occupational study conducted in Italy showed a significant positive association between PFOA and ALT levels among workers exposed to PFOA (coefficient, 0.116; 95% CI: 0.054, 0.177; *p* < 0.01) (Costa et al. 2009). Notably, these results are also consistent with data coming from a general population survey (mean ± SE linear regression coefficients with ALT: PFOA, 1.86 ± 0.62, *p* = 0.005; PFOS, 1.01 ± 0.53, *p* = 0.066) where background concentrations of PFOA are much lower than those reported here (Lin et al. 2010). Only one previous study of this same population reported no association with ALT (regression coefficient = –0.00416, *p* = 0.65); however, the observation was based on a much smaller sample (most of whom were likely to be included in the present analysis) and based on figures coming from models including non-log-transformed exposure or outcome variables (Emmett et al. 2006).

Evidence for an association between PFOA and PFOS and GGT is not so clear. Although there was some suggestion of an association in the linear regression models with PFOA, it was not replicated in logistic regression models. The instability of linear regression coefficients (which are very sensitive to the inclusion of additional covariates) might be due to a confounding effect of diet, or residual confounding of alcohol consumption, which causes a direct increase of GGT. Finally, the absence of any trend across districts—even though there are large contrasts in between-district mean exposures—might be indicative of some confounding factor acting at the individual level. As shown recently, GGT levels are positively associated with alcohol and meat intake after adjusting for all potential non-dietary confounders (Lee et al. 2004). To support this, there is indirect evidence from an occupational study that reported a significant association between ln-GGT and ln-PFOA in models adjusted by ln-age, ln-BMI, and ln-alcohol (mean ± SE coefficient, 0.033 ± 0.017, *p* = 0.05), which drops if BMI is replaced by ln-tryglicerides (a proxy for diet) in the model (coefficient, 0.010 ± 0.016, *p* = 0.55) (Olsen and Zobel 2007).

PFOA concentration has a positive association with direct bilirubin up to 40 ng/mL, followed by a decrease of bilirubin levels after this peak. A negative association between PFOA or PFOS and direct bilirubin has been observed in some occupational studies (Costa et al. 2009; Olsen and Zobel 2007; Sakr et al. 2007b), which might be because they used mainly subjects in the higher range of exposure, missing the first part of an inverse U-shaped curve. The figures of the between-district comparison might reflect the fact that the means of the four highest districts are well above the value of 30 ng/mL and may account for a larger portion of the observed association above that value, or this may be evidence of some geographic confounding.

The hepatotoxic effect of PFOA in rodents leading to liver enlargement and altered hepato-cyte histology (Qazi et al. 2010) appears to be mediated in part by PPAR-α agonism, leading to altered expression of the genes involved in peroxisome proliferation, cell cycle control, and apoptosis (Lau et al. 2007). Although different mechanisms also play a role in humans (Bjork et al. 2011), human cellular responses do predict a PPAR-α response (Rakhshandehroo et al. 2009; Yang et al. 2008). In the human population data presented here, the association of PFOS concentration with increasing trans-aminase is almost as prominent as the association for PFOA, and human laboratory cell data indicate relatively lower response of liver cells to PFOS than to PFOA (Wolf et al. 2008).

The consistency and significance of associations of ALT end points outside the normal range with PFOA serum concentration suggest a true association with an underlying hepatotoxic effect in humans. However, only a small proportion of end points fell outside the normal value range, making the observed results more difficult to interpret in terms of human health risk. In particular, it is not clear if this small increase in ALT levels can lead to clinically diagnosable conditions in the future, or if this effect is reversible (i.e., if it is reduced after removal of the exposure to PFOA). Finally, results coming from this study need to be read in the context of health effect of PFAAs on humans, but data cannot be directly used for estimating single-subject damage in relation to exposure.

The main limitation of the present study is the cross-sectional design, which makes causal inference particularly difficult. However, the consistency of the findings with previous literature—in particular the association with ALT—reinforces the hypothesis of a true association. Also, self-reported data of lifestyle characteristics being strongly associated with the exposures of interest can hamper a correct adjustment for potential confounders, which might be of particular relevance given the small magnitude of the observed associations. However, this study is the largest reported so far on this topic on a population-based sample of residents exposed to relatively high concentrations of PFOA.

## Conclusions

A small but clear linear association between PFOA and PFOS serum concentrations and ALT, a marker of hepatocellular injury, was observed in this large population-based sample of individuals with exposure to PFAAs. These results are consistent with previous findings and warrant further investigation, in particular on the potential health consequence of long-term exposure and potential -accumulation of damage.

## Supplemental Material

(172 KB) PDF
