# Supplementary material for: Serum Perfluorooctanoate (PFOA) and Perfluorooctane Sulfonate (PFOS) Concentrations and Liver Function Biomarkers in a Population with Elevated PFOA Exposure
Source: Environ Health Perspect. 2012 Jan 30;120(5):655–60. doi: 10.1289/ehp.1104436 (PMC3346788; doi:10.1289/ehp.1104436)
Supplement: (172 KB) PDF [file ehp.1104436.s001.pdf]

Supplemental Material

Serum Perfluorooctanoate (PFOA) and Perfluorooctane Sulfonate (PFOS) Concentrations and Liver Function Biomarkers in a Population with Elevated PFOA Exposure

Valentina Gallo[1,2], Giovanni Leonardi[1], Bernd Genser[3,4], Maria-Jose Lopez-Espinosa[1], Stephanie J Frisbee[5], Lee Karlsson[1], Alan M Ducatman[6], Tony Fletcher[1]

[1]Social and Environmental Health Research (SEHR), London School of Hygiene and Tropical Medicine, London, UK

[2]School of Public Health, Imperial College London, London, UK

[3]Mannheim Institute of Public Health, Social and Preventive Medicine, University of Heidelberg, Heidelberg, Germany

[4]Instituto de Saúde Coletiva, Federal University of Bahia, Salvador, Brazil

[5]Department of Community Medicine and Center for Cardiovascular and Respiratory Sciences, West Virginia University School of Medicine, Morgantown, WV, USA

[6]Department of Community Medicine, West Virginia University School of Medicine, West Virginia, USA

Detail of between- and within-regression models

Water district data available in the C8 Health Project questionnaire data were considered: using the geocoded locations of the address, combined with a detailed mapping of streets covered by each water districts piped water supplies, geocoded residences could be assigned a water district code. These analyses were restricted to those living in the six contaminated districts (Little Hocking Water Association of Ohio; City of Belpre, Ohio; Tupper Plains–Chester District of Ohio; Village of Pomeroy, Ohio; Lubeck Public Service District of West Virginia; Mason County Public Service District of West Virginia) at the time of the survey (n=26,777). For each water districts, on the ln-transformed scale, a mean PFOA value and a deviation from the mean for each individual was calculated as the difference between the individual level and the water district mean. Regression coefficients with relative standard errors (SE) and p-values were calculated for the association within water district and between water districts with both the mean ln-PFOA values, and the individual deviations, in a fully adjusted linear regression model. The significance of the difference between these within and between water district coefficients was also assessed. Models also included a random effect at the water district level.

Formal model description:

To estimate within and between water district (i=1,..., 6) coefficients relating log serum PFOA in individual j in that district ($x_{i,j}$) to numerical outcomes ($y_{i,j}$), we fit the model:

$$y_{i,j} = a + \beta_w(d_{i,j}) + \beta_b\bar{x}_i + \{\text{covariate terms}\} + \alpha_i + \varepsilon_{i,j} \quad ,$$

$$Where\ d_{i,j} = (x_{i,j} - \bar{x}_i), \alpha_i \sim N(0,\sigma_b^2), and\ \varepsilon_{i,j} \sim N(0,\sigma_w^2)$$

To test the hypothesis $\beta_w = \beta_b$, we re-parameterised this relationship writing $\beta_{diff} = \beta_w - \beta_b$, giving:

$$E(y) = a + \beta_w(x_{i,j}) + \beta_{difference}\bar{x}_i + \{\text{covariate terms}\}$$

We used the Wald test for $\beta_{diff} = 0$ as a test for $\beta_w = \beta_b$.

For dichotomous outcomes we fit analogous logistic models, except that instead of fitting a random effect at water district level, which was computationally cumbersome, we used a sandwich (Huber-White) estimator of variance clustering by water district.
